# Dimension reduction and shrinkage methods for high dimensional disease risk scores in historical data

**DOI:** 10.1186/s12982-016-0047-x

**Published:** 2016-04-05

**Authors:** Hiraku Kumamaru, Sebastian Schneeweiss, Robert J. Glynn, Soko Setoguchi, Joshua J. Gagne

**Affiliations:** Division of Pharmacoepidemiology and Pharmacoeconomics, Department of Medicine, Brigham and Women’s Hospital, Harvard Medical School, 1620 Tremont Street (Suite 3030), Boston, MA 02120 USA; Department of Healthcare Quality Assessment, Graduate School of Medicine, The University of Tokyo, 7-3-1 Hongo, Bunkyo-ku, Tokyo, 113-8654 Japan; Duke Clinical Research Institute, Duke University, 2400 Pratt Street, Durham, NC 27705 USA

**Keywords:** High dimensional propensity score, Disease risk score, Historical data, Shrinkage, Comparative study, Confounding

## Abstract

**Background:**

Multivariable confounder adjustment in comparative studies of newly marketed drugs can be limited by small numbers of exposed patients and even fewer outcomes. Disease risk scores (DRSs) developed in historical comparator drug users before the new drug entered the market may improve adjustment. However, in a high dimensional data setting, empirical selection of hundreds of potential confounders and modeling of DRS even in the historical cohort can lead to over-fitting and reduced predictive performance in the study cohort. We propose the use of combinations of dimension reduction and shrinkage methods to overcome this problem, and compared the performances of these modeling strategies for implementing high dimensional (hd) DRSs from historical data in two empirical study examples of newly marketed drugs versus comparator drugs after the new drugs’ market entry—dabigatran versus warfarin for the outcome of major hemorrhagic events and cyclooxygenase-2 inhibitor (coxibs) versus nonselective non-steroidal anti-inflammatory drugs (nsNSAIDs) for gastrointestinal bleeds.

**Results:**

Historical hdDRSs that included predefined and empirical outcome predictors with dimension reduction (principal component analysis; PCA) and shrinkage (lasso and ridge regression) approaches had higher c-statistics (0.66 for the PCA model, 0.64 for the PCA + ridge and 0.65 for the PCA + lasso models in the warfarin users) than an unreduced model (c-statistic, 0.54) in the dabigatran example. The odds ratio (OR) from PCA + lasso hdDRS-stratification [OR, 0.64; 95 % confidence interval (CI) 0.46–0.90] was closer to the benchmark estimate (0.93) from a randomized trial than the model without empirical predictors (OR, 0.58; 95 % CI 0.41–0.81). In the coxibs example, c-statistics of the hdDRSs in the nsNSAID initiators were 0.66 for the PCA model, 0.67 for the PCA + ridge model, and 0.67 for the PCA + lasso model; these were higher than for the unreduced model (c-statistic, 0.45), and comparable to the demographics + risk score model (c-statistic, 0.67).

**Conclusions:**

hdDRSs using historical data with dimension reduction and shrinkage was feasible, and improved confounding adjustment in two studies of newly marketed medications.

**Electronic supplementary material:**

The online version of this article (doi:10.1186/s12982-016-0047-x) contains supplementary material, which is available to authorized users.

## Background

Comparative effectiveness and safety assessments of newly marketed medical products in routine care are now widely conducted using large databases, including administrative claims databases and electronic health record data [1–3]. The numbers of potential confounders available for studies in these databases are large, and the utility of standard multivariable regression approaches can be limited especially when the number of study outcomes is small [4]. Propensity scores (PSs) have been used to circumvent this problem by modeling the exposure instead of the outcome [5–7]. In the presence of many potential confounders or proxies thereof, an algorithm for automated confounder selection may reduce bias beyond adjustment for variables pre-specified by researchers in studies using administrative claims databases [8]. The high dimensional PS (hdPS) algorithm generates, selects, and incorporates into a PS model the top potential confounders from thousands of empirically-identified diagnosis, procedure, and drug codes based on their strength of association with the outcome and exposure. However, for a new medical product in its early marketing phase, the hdPS approach can be limited due to evolving propensities over time and small numbers of exposed patients and outcomes [9]. Small number of outcomes can lead to unstable estimates of the outcome-covariate associations, and to residual confounding [10].

Historically derived disease risk scores (DRSs) may be a useful alternative to PSs in this setting. Although the number of individuals exposed to the new drug and the number of outcomes may be limited in the early marketing phase, there often exist many individuals exposed to the comparator product in the period preceding market entry of the new drug. Using a historical cohort to fit a DRS model and then applying the model as a prediction rule to estimate disease risk for patients using the drugs of interest after the market entry enables adjustment for a large number of potential confounders without having to fit a model in the study cohort [9]. While DRSs offer similar dimension reduction benefits as PSs and also have an important balancing property distinct from that of the PS for alternatively treated patients [11], empirical selection and inclusion of hundreds of potential confounders into the DRS estimation model will lead to over-fitting in the historical cohort and reduced predictive performance in the study cohort. In order to stably estimate historical high-dimensional DRSs (hdDRSs) with large numbers of variables, we propose the use of dimension reduction via principal component analysis and shrinkage with ridge and lasso regression. These techniques have been used often for prediction modeling in genetic epidemiology [12–14], but less frequently in clinical and pharmaco-epidemiology.

The objective of this study is to compare different approaches for hdDRS estimation in the historical comparator drug cohort for confounding adjustment in the concurrent study cohort of new and comparator drug initiators. We use two case studies to compare the methods: warfarin versus dabigatran on major hemorrhagic events and cyclooxygenase-2 inhibitor (coxibs) versus non-selective non-steroidal anti-inflammatory drugs (nsNSAIDs) on gastrointestinal (GI) bleeding events.

## Methods

### Data sources, study cohorts, and outcomes

#### Dabigatran example

**Fig. 1:**
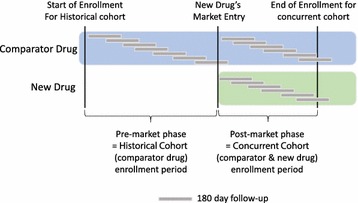
Patient enrollment and follow-up in the example studies

The dabigatran versus warfarin study was conducted using the United Healthcare claims database. As the concurrent study cohort, we identified patients 18 years or older, who initiated warfarin or dabigatran after October 2010, when dabigatran came into the market, through June 2012 (Fig. 1). Cohort entry was defined by the first prescription of medications which was preceded by 365 days of absence of either drug. We required patients to have a diagnosis of atrial fibrillation as defined by International Classification of Diseases, Ninth Revision, Clinical Modification (ICD-9-CM) code 427.31 anywhere on record prior to initiation, and excluded patients with prior diagnoses or procedures related to valvular heart disease (Additional file 1: Table S1), venous thromboembolism, or end stage renal disease [15] in the 365 days before cohort entry. Historical cohort of warfarin initiators consisted of patients who initiated warfarin between October 2008 and September 2009, with the same inclusion criteria. We followed patients for 180 days after cohort entry for occurrence of major hemorrhagic events, including intracranial hemorrhage, upper GI hemorrhage, lower and unspecified GI bleed, urogenital bleed, and other major bleeds captured using discharge diagnoses and procedure codes (listed in the Additional file 1: Table S2). Based on the results reported in the clinical trial, we used a relative risk of 0.93 for dabigatran against warfarin as a benchmark [16], knowing that this is a target with certain uncertainty.

#### Coxibs example

The coxibs versus nsNSAIDs study was conducted using the Pennsylvania Pharmaceutical Assistance Contract for Elderly (PACE) program database linked to Medicare claims data [17]. In the concurrent study cohort, we included patients who initiated any nsNSAIDs, celecoxib, or rofecoxib between January 1999 and December 31, 2002. We defined initiation as the first dispensing of one of these drugs following a period of at least 6 months of absence, and designated the date of dispensing as the cohort entry date. nsNSAIDs initiators with entry dates between January 1, 1997 through Dec 31, 1998, before celecoxib came on to the market, comprised the historical cohort. We followed patients for 180 days after their entry for occurrence of GI bleeds, captured using a validated claims-based algorithm [18]. Based on the reports from randomized clinical trials and meta-analyses, we used relative risk of 0.50–0.64 as a benchmark [19–22].

The studies were approved by the review board of our institution and were carried out under an established data use agreement.

### High-dimensional disease risk score estimation using historical data

We used the historical cohorts to conduct hdDRS estimation via three steps, similarly to the hdPS algorithm [8]: (1) empirical variable identification; (2) variable prioritization; and (3) model specification. The models were then used in a fourth step to estimate the hdDRS in the concurrent cohorts.

#### Variable identification and prioritization

In each of the data dimensions of inpatient diagnoses (ICD-9-CM), outpatient diagnoses (ICD-9-CM), inpatient and outpatient procedures (ICD-9-CM and Current Procedural Terminology, 4th edition), drug prescriptions (generic names), and nursing home diagnoses (ICD-9-CM), we identified the 300 most prevalent codes. Prevalence was calculated as the proportion of eligible patients in the historical cohorts with the code on record at least once in the 365-day period immediately preceding each patient’s cohort entry date.

We assessed the association of each code with the outcome by fitting univariable logistic regression models predicting the study outcomes. The total of 1500 (300 × 5 data dimensions) codes were ranked based on the estimated likelihood of these models, and the 500 variables with the highest likelihoods were selected.

#### Model specification

We developed 14 multivariable logistic regression models to predict the outcome of interest in the historical cohorts using combinations of the following data components: (a) demographic variables; (b) predefined risk factors for the outcome (listed in Additional file 1: Table S3); (c) established risk prediction scores: the HAS-BLED [23] score for major hemorrhagic events in the dabigatran study and combined comorbidity score [24] for gastrointestinal bleeds in the coxib study (as there is no established risk score for GI bleeds in nsNSAIDs or coxibs users, we utilized the combined comorbidity score, which captures overall comorbidity burden among older individuals); and (d) the 500 empirically-selected codes.

We used dimension reduction via principal component analysis (PCA) and shrinkage with lasso and ridge regression in some models to reduce overfitting. For models involving principal components, we first conducted the PCA to identify 30 principal components based on the 500 empirically-selected codes and then included either the top 10 or all 30 components in the DRS models. We chose a maximum of 30 principal components to maintain the event-to-variable ratio above 5 in the model with demographic variables. Ridge and lasso regressions were conducted using the ‘glmnet’ package in R [25]. Shrinkage tuning parameter, lambda, was selected through 10-fold cross validation minimizing the deviance of the prediction model in the testing sample.

To evaluate the added value of the empirically-selected codes, we constructed models including demographic variables, predefined variables, and established risk scores with and without the empirically selected codes. To evaluate the effect of PCA and shrinkage, a model including the 500 empirically-identified codes without dimension reduction or shrinkage was also created.

The coefficients from the 14 different models developed in the historical cohort were applied to the patients in the concurrent cohort to predict the baseline probability (DRS) of the outcome for each patient at the index date.

### Statistical analysis and model evaluation

We report baseline characteristics of the historical and concurrent cohorts by presenting proportions and means of the demographic and predefined variables as well as the established risk scores. We evaluated the predictive performances of the DRS models by assessing discrimination (c-statistics) and calibration [Hosmer–Lemeshow (H–L) test] of the DRSs applied to the concurrent cohort. The c-statistics and H–L test statistics were estimated in the new drug and the comparator drug initiators separately. As an additional visual aid, we also present calibration plots for a selected set of models in the dabigatran study. We also report the nominal c-statistics of the models in the historical cohort and their 95 % confidence intervals, as well as the c-statistics from 10-fold cross validation to evaluate over-fitting of the models in the historical cohorts. We assessed the correlation between the number of events per variable in the DRS models and the gap between nominal and cross-validated c-statistcs by Spearman’s rank correlation. Finally, we estimated the DRS-adjusted relative odds of the study outcome for new drug initiation compared to comparator drug initiation using logistic regression stratified by DRS deciles. We selected the use of stratification over other methods such as matching so as to compare the effect estimates among the same population. All analyses were conducted using SAS 9.3 (SAS Institute, Inc., Cary, NC) and R version 3.0.2 (Vienna, Austria).

## Results

Population characteristics and observed numbers of study outcomes in the two example studies are presented in Tables 1 and 2.

**Table 1:** Baseline characteristics and observed risk of major hemorrhagic events within 180 days of the warfarin and dabigatran initiators in the historical and concurrent cohorts

Variable	Historical cohort (Oct 1 2008–Sept 30 2010^a^)	Concurrent cohort (Oct 1 2010–June 30 2012^a^)	
	Warfarin (N = 10,014)	Warfarin (N = 5360)	Dabigatran (N = 3874)
Age, mean (SD)	63.9 (11.5)	64.6 (11.8)	61.8 (11.5)
Female, n (%)	2974 (29.7)	1686 (31.5)	1040 (26.8)
Nursing home stay, n (%)	376 (3.8)	282 (5.3)	3815 (1.5)
Num. of medications, mean (SD)	11.0 (6.4)	11.2 (6.5)	10.1 (5.9)
Num. of physician visits, mean (SD)	15.8 (18.9)	17.1 (20.8)	12.9 (12.4)
At least 1 hospitalization, n (%)	4911 (49.0)	2686 (50.1)	1594 (41.2)
Use of proton pump Inhibitors, n (%)	1864 (18.6)	1092 (20.4)	689 (17.8)
Use of antiplatelets, n (%)	1155 (11.5)	611 (11.4)	399 (10.3)
Use of nsNSAIDs, n (%)	2231 (22.3)	1105 (20.6)	842 (21.7)
ICH hosp., n (%)	25 (0.2)	20 (0.4)	5 (0.1)
GI bleed hosp., n (%)	69 (0.7)	40 (0.7)	16 (0.4)
GI bleed, n (%)	457 (4.6)	262 (4.9)	152 (3.9)
Peripheral artery disease, n (%)	1190 (11.9)	747 (13.9)	362 (9.3)
Anemia, n (%)	1412 (14.1)	855 (16.0)	400 (10.3)
Chronic liver disease, n (%)	217 (2.2)	136 (2.5)	89 (2.3)
Chronic kidney disease, n (%)	1524 (15.2)	1046 (19.5)	452 (11.7)
Alcohol addiction, n (%)	247 (2.5)	116 (2.2)	85 (2.2)
Drug abuse, n (%)	106 (1.1)	53 (1.0)	46 (1.2)
HAS-BLED mean (SD)	2.0 (1.5)	2.1 (1.6)	1.8 (1.4)
Major hemorrhagic event, n (%)	254 (2.5 %)	129 (2.4 %)	49 (1.3 %)

*GI bleed* gastrointestinal bleeding, *HAS-BLED* HAS-BLED hemorrhage risk score, *hosp.* hospitalizations, *Num.* number

^a^Enrollment period

**Table 2:** Baseline characteristics and observed risk of gastrointestinal bleeds within 180 days of the non-selective nonsteroidal anti-inflammatory drugs and cyclooxygenase-2 inhibitors initiators in the historical and concurrent cohorts

Variable	Historical cohort (Jan 1 1997–Dec 31 1998^a^)	Concurrent cohort (Jan 1 1999–Dec 31 2001^a^)	
	nsNSAIDs (N = 28,533)	nsNSAIDs (N = 15,930)	Coxibs (N = 31,875)
Age, mean (SD)	78.6 (6.8)	78.9 (6.9)	80.5 (6.8)
White, n (%)	26,535 (93.0)	14,450 (90.7)	30,583 (95.9)
Female, n (%)	24,011 (84.2)	13,287 (83.4)	27,812 (87.3)
Nursing home stay, n (%)	1757 (6.2)	989 (6.2)	2781 (8.7)
Num. of medications, mean (SD)	9.4 (5.1)	9.7 (5.2)	10.5 (5.4)
Num. of physician visits, mean (SD)	9.7 (6.9)	9.3 (6.7)	9.8 (6.7)
At least 1 hospitalization, n (%)	8514 (29.8)	2267 (28.0)	10,497 (32.9)
Use of warfarin, n (%)	2148 (7.5)	1212 (7.6)	4509 (14.1)
Use of gastroprotective drugs, n (%)	9059 (31.7)	4560 (28.6)	12,238 (38.4)
Use of corticosteriods, n (%)	2445 (8.6)	1435 (9.0)	3265 (10.2)
Use of clopidogrel, n (%)	671 (2.4)	785 (4.9)	2148 (6.7)
Rheumatoid arthritis, n (%)	1215 (4.3)	519 (3.3)	1721 (5.4)
Congestive heart failure, n (%)	5892 (20.6)	3295 (20.7)	7864 (24.7)
Osteoarthritis, n (%)	13,044 (45.7)	6645 (41.7)	17,812 (55.9)
Peripheral vascular disease, n (%)	5173 (18.1)	3192 (20.0)	7387 (23.2)
GI bleeding, n (%)	1352 (4.7)	788 (4.9)	1972 (6.2)
Chronic kidney diseases, n (%)	965 (3.4)	684 (4.3)	1275 (4.0)
Carotid artery disease, n (%)	11,551 (40.5)	6460 (40.6)	13,944 (43.8)
Combined comorbidity score >2, n (%)	7266 (25.5)	4170 (26.2)	10,051 (31.5)
GI bleed, n (%)	201 (0.7 %)	87 (0.6 %)	189 (0.6 %)

*Coxibs* cyclooxygenase-2 inhibitors, *GI bleed* gastrointestinal bleeding, *nsNSAIDs* non-selective nonsteroidal anti-inflammatory drugs, *Num.* number

^a^Enrollment period

### Dabigatran example

We identified 10,014 patients initiating warfarin during the historical period, and 5360 warfarin initiators and 3874 dabigatran initiators during the concurrent period. Of these, 254 (2.5 %) historical warfarin initiators experienced hemorrhagic events during the 180 days after initiation, while 129 (2.4 %) and 49 (1.3 %) patients had hemorrhagic events in the concurrent warfarin and dabigatran initiators, respectively. In general, dabigatran initiators were younger and had fewer comorbidities than warfarin initiators in both periods. The unadjusted OR for hemorrhagic events within 180 days was 0.52 [95 % confidence interval (CI) 0.37, 0.72].

### Coxibs example

We identified 28,533 nsNSAIDs initiators in the historical cohort, and 15,930 nsNSAID and 31,875 coxib initiators in the concurrent cohort. During the 180 days of follow-up, we identified 201 (0.7 %), 87 (0.6 %) and 189 (0.6 %) GI bleeding events in the historical nsNSAID, concurrent nsNSAID, and coxib initiators, respectively. Coxib initiators were older and had more comorbidities, higher frequencies of health service use, and more medication use as compared to nsNSAID initiators in either period. The unadjusted OR for GI bleeds within 180 days was 1.09 (95 % CI 0.84, 1.40).

### Discrimination and calibration

Discrimination and calibration statistics of the 14 DRS models in the historical cohort as well as in the concurrent cohort are presented in Tables 3 and 4. As the number of events-per-variable included in the DRS model decreased, the gap between the nominal and the cross validated c-statistics in the historical cohort increased, when no shrinkage was applied (Spearman correlation coefficients: −0.89 for dabigatran study; −0.98 for coxib study). Smaller events-per-variable ratios were also associated with higher HL statistics in the comparator drug initiators in the concurrent cohort, when no shrinkage is applied.

**Table 3:** Predictive performance of the disease risk score (DRS) models in the warfarin versus dabigatran historical and concurrent cohorts

Num.	Model data component and dimension reduction/shrinkage method	EPV^b^	Historical cohort			Concurrent cohort					
			Warfarin initiators			Warfarin initiators			Dabigatran initiators		
			c-stat	95 % CI	x-valid. c-stat	c-stat	95 % CI	HL (*P* value)	c-stat	95 % CI	HL (P value)
1	Demo (age + sex)	127.0	0.58	0.55, 0.62	0.59	0.59	0.54, 0.63	2.7 (0.95)	0.68	0.61, 0.75	28.1 (<0.01)
2	Demo + score^a^	84.7	0.61	0.57, 0.64	0.60	0.62	0.57, 0.67	5.6 (0.69)	0.70	0.63, 0.77	24.6 (<0.01)
3	Demo + score + predef.	16.9	0.64	0.60, 0.67	0.61	0.61	0.56, 0.66	7.5 (0.48)	0.69	0.63, 0.76	23.0 (<0.01)
4	Demo + cov500	0.5	0.86	0.84, 0.89	0.61	0.54	0.49, 0.60	384 (<0.01)	0.56	0.48, 0.64	126 (<0.01)
5	Demo + PCA(10)	21.2	0.67	0.64, 0.71	0.65	0.66	0.61, 0.71	5.2 (0.73)	0.68	0.61, 0.75	27.0 (<0.01)
6	Demo + PCA(30)	7.9	0.69	0.65, 0.72	0.63	0.63	0.59, 0.68	10.2 (0.25)	0.64	0.56, 0.72	18.5 (0.02)
7	Demo + score + PCA(10)	19.5	0.67	0.64, 0.71	0.64	0.64	0.59, 0.69	9.8 (0.28)	0.67	0.60, 0.73	18.1 (0.02)
8	Demo + score + PCA(30)	7.7	0.69	0.65, 0.72	0.63	0.62	0.57, 0.67	10.1 (0.26)	0.64	0.56, 0.72	17.6 (0.02)
9	Demo + predef. + score + PCA(10)	10.2	0.69	0.66, 0.72	0.64	0.64	0.59, 0.69	16.0 (0.04)	0.66	0.59, 0.73	20.3 (0.01)
10	Demo + predef. + score + PCA(30)	5.6	0.71	0.67, 0.74	0.63	0.63	0.58, 0.68	21.1 (0.01)	0.66	0.59, 0.73	18.9 (0.02)
11	Ridge (Demo + predef. + score + cov500)	0.5	0.83	0.81, 0.86	0.69	0.63	0.59, 0.68	12.3 (0.14)	0.63	0.55, 0.71	19.5 (0.01)
12	Ridge (Demo + predef. + score + PCA(30))	5.6	0.71	0.68, 0.74	0.65	0.64	0.58, 0.68	14.9 (0.06)	0.67	0.59, 0.75	25.5 (<0.01)
13	Lasso (Demo + predef. + score + cov500)	0.5	0.72	0.69, 0.75	0.63	0.64	0.59, 0.68	10.8 (0.21)	0.66	0.59, 0.73	27.7 (<0.01)
14	Lasso (Demo + predef. + score + PCA(30))	5.6	0.70	0.67, 0.73	0.65	0.65	0.61, 0.70	10.7 (0.22)	0.67	0.59, 0.74	19.5 (0.01)

*CI* confidence interval, *Demo* demographic variables, *HL* Hosmer–Lemeshow test statistics, *Num.* model number, *predef.* predefined variables, *PCA*(*10*) top 10 components from principal component analysis, *PCA*(*30*) top 30 components from principal component analysis, *c-stat* c-statistics, *x-valid.* 10-fold cross-validated

^a^Score = HAS-BLED score [23]

^b^Event per variable: ratio between the number of outcomes and number of variables included in the DRS model

**Table 4:** Predictive performance of the disease risk score (DRS) models in the cyclooxygenase-2 inhibitor versus non-selective non-steroidal anti-inflammatory drugs in historical and concurrent cohorts

Num.	Model data component and dimension reduction/shrinkage method	EPV^c^	Historical cohort			Concurrent cohort					
			nsNSAID initiators			nsNSAID initiators			coxib initiators		
			c-stat	95 % CI	x-valid. c-stat	c-stat	95 % CI	HL (P value)	c-stat	95 % CI	HL (P value)
1	Demo (age + sex + race)	40.2	0.64	0.60, 0.67	0.63	0.64	0.59, 0.70	9.6 (0.30)	0.59	0.55, 0.63	24.4 (<0.01)
2	Demo + score^a^	33.5	0.66	0.62, 0.69	0.65	0.67	0.61, 0.72	9.6 (0.30)	0.65	0.62, 0.68	34.2 (<0.01)
3	Demo + score + predef.	7.2	0.70	0.67, 0.74	0.66	0.66	0.59, 0.72	12.0 (0.15)	0.62	0.58, 0.66	41.2 (<0.01)
4	Demo + cov500	0.4	0.96	0.95, 0.97	0.71	0.45	0.38, 0.51	>999 (<0.01)	0.48	0.43, 0.52	>999 (<0.01)
5	Demo + PCA(10)	13.4	0.72	0.68, 0.75	0.68	0.66	0.60, 0.72	10.4 (0.24)	0.63	0.59, 0.67	32.1 (<0.01)
6	Demo + PCA(30)	5.7	0.75	0.72, 0.79	0.69	0.66	0.61, 0.72	12.4 (0.13)	0.65	0.62, 0.69	56.5 (<0.01)
7	Demo + score + PCA(10)	12.6	0.72	0.68, 0.75	0.68	0.66	0.60, 0.72	12.1 (0.15)	0.62	0.58, 0.66	34.8 (<0.01)
8	Demo + score + PCA(30)	5.6	0.75	0.72, 0.79	0.69	0.66	0.60, 0.72	12.6 (0.12)	0.65	0.61, 0.69	58.5 (<0.01)
9	Demo + predef. + score + PCA(10)	5.3	0.74	0.70, 0.77	0.67	0.65	0.59, 0.72	21.7 (0.01)	0.60	0.56, 0.64	74.3 (<0.01)
10	Demo + predef. + score + PCA(30)	3.5	0.78	0.75, 0.81	0.70	0.66	0.60, 0.72	23.6 (< 0.01)	0.63	0.58, 0.67	102.6 (<0.01)
11	Ridge (Demo + predef. + score + cov500)	0.4	0.92	0.90, 0.93	0.77	0.55	0.48, 0.62	77.3 (<0.01)	0.59	0.54, 0.63	172.9 (<0.01)
12	Ridge (Demo + predef. + score + PCA(30))	3.5	0.77	0.74, 0.80	0.71	0.67	0.61, 0.72	11.9 (0.16)	0.64	0.60, 0.68	35.2 (<0.01)
13	Lasso (Demo + predef. + score + cov500)	0.4	0.93	0.91, 0.94	0.72^b^	0.53	0.46, 0.60	409 (<0.01)	0.57	0.52, 0.61	578.8 (<0.01)
14	Lasso (Demo + predef. + score + PCA(30))	3.5	0.78	0.74, 0.81	0.72	0.67	0.61, 0.73	9.1 (0.33)	0.65	0.61, 0.69	38.3 (<0.01)

*c-stat* c-statistics, *coxibs* cyclooxygenase-2 inhibitors, *Demo* demographic variables, *HL* Hosmer–Lemeshow test statistics, *nsNSAIDs* non-selective nonsteroidal anti-inflammatory drug, *Num.* model number, *PCA*(*10*) top 10 components from principal component analysis, *PCA*(*30*) top 30 components from principal component analysis, *predef.* predefined variables, *x-valid.* 10-fold cross-validated

^a^Score = combined comorbidity score [24]

^b^Average of 3 of 10 which reached convergence in the 10-fold cross-validation, the rest did not reach convergence

^c^Event per variable: ratio between the number of outcome and number of variables included in the model

The model with the 500 empirical covariates without dimension reduction or shrinkage (model 4) had the largest gap between the nominal and cross-validated c-statistic in the historical cohort, and also had the lowest c-statistics and highest HL test statistics when applied to the concurrent cohort. Many of the models with empirically identified variables (models 5–14) had higher cross-validated c-statistics in the historical cohort compared to the models without empirically identified variables (models 1–3), suggesting an improvement in the predictive performance with the addition of these variables, although this did not always lead to better c-statistics in the concurrent cohort. In the dabigatran example, the cross-validated c-statistics corresponded well with the c-statistics estimated in the concurrent cohorts for most models except for the unreduced model (model 4). In the coxibs example, the cross-validated c-statistics were generally higher than the concurrent cohort c-statistics. Models 11 and 13 in the coxib example had extremely high c-statistics in the historical cohort (both nominal and cross-validated, although with large gaps), with very low c-statistics in the concurrent cohort, indicating extreme optimism likely due to overfitting. HL test statistics for the comparator initiators were not significant for many of the models with higher c-statistics. In the new drug initiators, HL test statistics were all statistically significant. The calibration plots for a selected set of models show that poor calibration for the dabigatran initiators is caused by overestimation of risk (Additional file 2: Figure S1).

### Estimated treatment effects

In both examples, stratification by deciles of DRSs with higher c-statistics and low HL-test statistics led to larger changes in the OR in the expected direction (Tables 5, 6). Stratification based on DRSs with either low c-statistics or high HL-statistics resulted in little change in the estimated ORs. In the dabigatran example, the largest adjustment in the expected direction was achieved by lasso + PCA model and demographics + 10 principal components model. In the coxib example, the largest reduction was achieved by the model with demographics and combined comorbidity score.

**Table 5:** The relative odds of major hemorrhagic events within 180 days for dabigatran initiators compared to warfarin initiators adjusted by DRS decile stratification

Model Num.	Model data component and dimension reduction/shrinkage method used	Adjusted by DRS deciles^b^	
		Odds ratio	95 % CI
	Crude	0.52	0.37, 0.72
1	Demo (age + sex + race)	0.58	0.41, 0.81
2	Demo + score^a^	0.60	0.43, 0.84
3	Demo + score + predef.	0.58	0.41, 0.81
4	Demo + cov500	0.53	0.38, 0.74
5	Demo + PCA(10)	0.64	0.45, 0.89
6	Demo + PCA(30)	0.62	0.44, 0.87
7	Demo + score + PCA(10)	0.61	0.44, 0.86
8	Demo + score + PCA(30)	0.61	0.44, 0.86
9	Demo + predef. + score + PCA(10)	0.60	0.43, 0.84
10	Demo + predef. + score + PCA(30)	0.60	0.43, 0.84
11	Ridge (Demo + predef. + score + cov500)	0.58	0.41, 0.81
12	Ridge (Demo + predef. + score + PCA(30))	0.61	0.44, 0.85
13	Lasso (Demo + predef. + score + cov500)	0.60	0.43, 0.85
14	Lasso (Demo + predef. + score + PCA(30))	0.64	0.46, 0.90

*CI* confidence interval, *Demo* demographic variables, *DRS* disease risk scores, *OR* odds ratios, *PCA*(*10*) top 10 components from principal component analysis, *PCA*(*30*) top 30 components from principal component analysis, *predef.* predefined variables

^a^Score = HAS-BLED score [23]

^b^Stratified by DRS decile indicators

**Table 6:** Relative odds of gastrointestinal bleeds within 180 days for cyclooxigenaze-2 inhibitor initiators compared to nsNSAIDs initiators adjusted by DRS decile stratification

Model Num.	Model data component and dimension reduction/shrinkage method used	Adjusted by DRS deciles^b^	
		Odds ratio	95 % CI
	Crude	1.09	0.84, 1.40
1	Demo (age + sex + race)	0.98	0.76, 1.27
2	Demo + score^a^	0.93	0.72, 1.20
3	Demo + score + predef.	0.97	0.75, 1.25
4	Demo + cov500	1.05	0.82, 1.36
5	Demo + PCA(10)	0.97	0.75, 1.25
6	Demo + PCA(30)	0.97	0.75, 1.25
7	Demo + score + PCA(10)	0.98	0.76, 1.26
8	Demo + score + PCA(30)	0.97	0.75, 1.25
9	Demo + predef. + score + PCA(10)	1.00	0.77, 1.29
10	Demo + predef. + score + PCA(30)	0.98	0.76, 1.27
11	Ridge (Demo + predef. + score + cov500)	1.04	0.80, 1.34
12	Ridge (Demo + predef. + score + PCA(30))	0.96	0.74, 1.24
13	Lasso (Demo + predef. + score + cov500)	1.05	0.82, 1.36
14	Lasso (Demo + predef. + score + PCA(30))	0.95	0.73, 1.22

*CI* confidence interval, *Demo* demographic variables (age, sex and race), *DRS* disease risk scores, *OR* odds ratios, *PCA*(*10*) top 10 components from principal component analysis, *PCA*(*30*) top 30 components from principal component analysis, *predef.* predefined variables

^a^Score = combined comorbidity score [24]

^b^Stratified by DRS decile indicators

## Discussion

The addition of empirically identified outcome predictors coupled with dimension reduction and shrinkage techniques in historical DRS development led to generally better predictive performance than DRS models without empirically identified predictors and clearly outperformed DRS models with the same empirical predictors that did not use dimension reduction or shrinkage techniques.

DRSs offer important advantages to other methods for assessing the comparative safety and effectiveness of new drugs in the early marketing setting. As with PSs, DRSs facilitate the incorporation of a large number of covariates for confounding adjustment. However, in the early marketing setting, few exposures and evolving prescribing patterns can limit the use of PSs. Developing a DRS in historical data and applying it to a concurrent cohort can overcome these limitations. Given that empirical variable selection has been shown to enhance confounding adjustment when included in PSs [8], inclusion of these potential confounding variables likely also enhances the ability of DRSs to mitigate confounding. Use of the DRS and its limitations are less well studied compared to PS [26, 27]. Unlike PS-based analyses where covariate balance can be easily assessed upon stratification, matching or weighting, prognostic balance cannot be readily assessed in DRS based analyses. Hansen has proposed the “dry-run” analysis for assessing prognostic balance [28], but it has not yet been formally evaluated. In addition, in the presence of strong correlation between the covariates and the exposure, DRS-based analyses may exaggerate the statistical significance of the effect measure, although later studies have shown that this is only problematic in the presence of extreme correlation that is rarely observed in practice [26, 29]. Limited investigation on the effect of misspecification of the DRS model have been conducted, and require further work.

Use of historically developed hdDRSs may be limited by two key factors. The first is over-fitting of the models in the historical cohort and reduced generalizability of the model to the concurrent cohort. This problem was exemplified by the worse performance of the unreduced model (model 4), in which 500 empirically identified covariates were included with no dimension reduction or shrinkage. High nominal c-statistics in the historical cohort but low c-statistics and extreme HL-statistics in the concurrent cohort demonstrated severe overfitting. For all models, the gap between the nominal and cross-validated c-statistics in the historical cohort is an indication of over-fitting and, as expected, the gaps were larger for the models with smaller event-per-variable ratios. The second key problem is the possibility of changes in the associations between the covariates and outcomes over time as populations and treatment strategies change. Any change in coding practice and clinical practice patterns between the historical and concurrent periods may lead to misspecification of the covariate-outcome association in the historically-developed DRS. This may happen, for example, if there is a procedure that is used to treat severer patients in the historical population, but are more often used to treat healthier patients in the concurrent cohort. We tried to indirectly evaluate this, in part, by assessing the discordance between the cross-validated c-statistics in the historical cohort and the c-statistics in the concurrent cohort, but it is impossible to completely disentangle the effect of overfitting and changes in the covariate-outcome relationship over time. The gap was not prominent for the models that used PCA, but present for most of the models that included the covariates without PCA even with shrinkage by lasso or ridge regression.

Because we evaluated the performance of the hdDRS models in two examples using two large administrative databases from the US, the results may not generalize to other study settings where the number of outcomes in the historical cohort is different, the number of potential confounders may be different, or where coding practices or clinical practice evolve in different patterns. Furthermore, we used the results of randomized trials as benchmarks against which to compare the performance of the hdDRS modeling strategies, but the results in observational studies may differ from randomized trials for several reasons other than incomplete confounding control. For example, in the dabigatran study example, the patients in our cohort were younger (mean age 71.6 years in the RE-LY trial vs. 64.6 years in the warfarin groups) and were more likely to be on proton pump inhibitors at baseline (13.8 % in the RE-LY trial vs. 20.4 % in the warfarin groups). In the presence of effect modification, this difference in patient characteristics (and of the effect modifier distribution) can lead to different relative risk estimates. In addition, factors such as differences in outcome definitions, outcome capture, and patient treatment adherence can lead to different estimates in routine care comparative effectiveness estimates and trials. It is therefore not surprising that even the odds ratio estimates closest to the benchmarks in our analyses did not exactly reach the benchmarks, and at the same time, some uncertainty may remain in our evaluation of the adjusted estimates from DRS stratification.

It is also important to highlight that while these high dimensional approaches try to maximize the use of the measured covariates for confounding control, they cannot account for unmeasured confounding. For hdDRS, the prediction of the models will be differentially affected in the new drug group versus the comparator drug group when the omitted variable is a confounder, leading to a biased relative risk estimate. Because we selected covariates to include in the model based on their univariate association with the outcome, it is possible that confounding from the interaction of two or more covariates are not captured in the models. Also, since our cohorts had limited numbers of patients and outcomes, the precision of estimates was limited, precluding meaningful statistical comparisons. Carefully designed simulation studies with high data dimensionality and incorporating covariate-outcome association changes over time that are reflective of the real-world may be needed to assess the true relative performances of these approaches. Lastly, we acknowledge the scarceness of prior evidence in the use of dimension reduction or shrinkage approaches in the context of DRSs. While our study showed a strong correlation between improved predictive performance from the use of these methods and estimated relative risk estimates that are closer to the expected values from the trials, further work is needed to investigate the optimal use of DRS methods for the purpose of confounding control.

We expect that the addition of different data sources, such as clinical registries or electronic medical record data could further enhance the performance of hdDRS approaches. As previously suggested, combining DRSs with PS, such as by matching on both PS and DRS, matching by the Mahalanobis distance, and subclassification by both PS and DRS [9, 30, 31], is a promising approach to obviate bias when one of the two models is misspecified, although the application of these methods has not been well studied in a high dimensional context and warrants further investigation. Finally, adaptive hdDRS approaches in the setting of prospective monitoring of new drugs, in which the historical cohort grows with the prospective accumulation of data, could mitigate the impact of changes in coding practice and clinical practice patterns on model misspecification. Inclusion of the concurrent comparator drug initiators may help reduce the effect of changing covariate-outcome patterns, but will need to be balanced with the challenges of developing the DRS in part of the study cohort as described by Hansen.

## Conclusions

hdDRSs developed using the historical comparator drug initiator cohort with dimension reduction and shrinkage methods was feasible for confounding adjustment in comparative studies using large administrative databases. This approach led to substantial improvement in the prediction of outcomes in the concurrent cohort over high-dimensional approaches that do not involve dimension reduction or shrinkage, while performing better than or at least as well as models based on investigator-defined variables only.

## Additional files

10.1186/s12982-016-0047-x Tables for codes used and predefined predictive factors for the two studies.
10.1186/s12982-016-0047-x Calibration Plots for Selected Models from dabigatran study.

## Abbreviations

CI: confidence interval
Coxibs: cyclooxygenase-2 inhibitor
DRS: disease risk score
GI: gastrointestinal
hdDRS: high dimensional disease risk score
hdPS: high dimensional propensity score
H–L: Hosmer–Lemeshow
ICD-9-CM: International Classification of Diseases, Ninth Revision, Clinical Modification
nsNSAIDs: non-steroidal anti-inflammatory drugs
OR: odds ratio
PACE: Pennsylvania Pharmaceutical Assistance Contract for Elderly
PCA: principal component analysis
PS: propensity score

## References

[1] Gagne JJ, Rassen JA, Choudhry NK, Bohn RL, Patrick AR, Sridhar G, Daniel GW, Liu J, Schneeweiss S (2014). Near-real-time monitoring of new drugs: an application comparing prasugrel versus clopidogrel. Drug Saf.

[2] Schneeweiss S, Gagne JJ, Glynn RJ, Ruhl M, Rassen JA (2011). Assessing the comparative effectiveness of newly marketed medications: methodological challenges and implications for drug development. Clin Pharmacol Ther.

[3] Schneeweiss S, Huybrechts KF, Gagne JJ (2013). Interpreting the quality of health care database studies on the comparative effectiveness of oral anticoagulants in routine care. Comp Eff Res.

[4] Harrell FE, Lee KL, Califf RM, Pryor DB, Rosati RA (1984). Regression modelling strategies for improved prognostic prediction. Stat Med.

[5] Rosenbaum PR, Rubin DB (1983). The central role of the propensity score in observational studies for causal effects. Biometrika.

[6] Rubin DB (1997). Estimating causal effects from large data sets using propensity scores. Ann Intern Med.

[7] Sturmer T, Joshi M, Glynn RJ, Avorn J, Rothman KJ, Schneeweiss S, Stürmer T, Joshi M, Glynn RJ, Avorn J, Rothman KJ, Schneeweiss S (2006). A review of the application of propensity score methods yielded increasing use, advantages in specific settings, but not substantially different estimates compared with conventional multivariable methods. J Clin Epidemiol.

[8] Schneeweiss S, Rassen JA, Glynn RJ, Avorn J, Mogun H, Brookhart MA (2009). High-dimensional propensity score adjustment in studies of treatment effects using health care claims data. Epidemiology.

[9] Glynn RJ, Gagne JJ, Schneeweiss S (2012). Role of disease risk scores in comparative effectiveness research with emerging therapies. Pharmacoepidemiol Drug Saf.

[10] Rassen JA, Glynn RJ, Brookhart MA, Schneeweiss S (2011). Covariate selection in high-dimensional propensity score analyses of treatment effects in small samples. Am J Epidemiol.

[11] Hansen BB (2008). The prognostic analogue of the propensity score. Biometrika.

[12] Pomeroy SL, Tamayo P, Gaasenbeek M, Sturla LM, Angelo M, McLaughlin ME, Kim JYH, Goumnerova LC, Black PM, Lau C, Allen JC, Zagzag D, Olson JM, Curran T, Wetmore C, Biegel JA, Poggio T, Mukherjee S, Rifkin R, Califano A, Stolovitzky G, Louis DN, Mesirov JP, Lander ES, Golub TR (2002). Prediction of central nervous system embryonal tumour outcome based on gene expression. Nature.

[13] Riedelsheimer C, Czedik-Eysenberg A, Grieder C, Lisec J, Technow F, Sulpice R, Altmann T, Stitt M, Willmitzer L, Melchinger AE (2012). Genomic and metabolic prediction of complex heterotic traits in hybrid maize. Nat Genet.

[14] Wang D, SalahElBasyoni I, Stephen Baenziger P, Crossa J, Eskridge KM, Dweikat I (2012). Prediction of genetic values of quantitative traits with epistatic effects in plant breeding populations. Heredity (Edinb).

[15] January CT, Wann LS, Alpert JS, Calkins H, Cleveland JC, Cigarroa JE, Conti JB, Ellinor PT, Ezekowitz MD, Field ME, Murray KT, Sacco RL, Stevenson WG, Tchou PJ, Tracy CM, Yancy CW. 2014 AHA/ACC/HRS guideline for the management of patients with atrial fibrillation: a report of the American College of Cardiology/American Heart Association Task Force on Practice Guidelines and the Heart Rhythm Society. J Am Coll Cardiol. 2014;64:e1–e76. 10.1016/j.jacc.2014.03.022 24685669

[16] Connolly S, Ezekowitz M, Reilly PA, Themeles E, Varrone J, Wang S, Alings M, Xavier D, Zhu J, Diaz R, Lewis BS, Darius H, Diener H, Joyner CD, Wallentin L, Committee RS (2009). Dabigatran versus warfarin in patients with atrial fibrillation. New.

[17] Solomon DH, Schneeweiss S, Glynn RJ, Kiyota Y, Levin R, Mogun H, Avorn J (2004). Relationship between selective cyclooxygenase-2 inhibitors and acute myocardial infarction in older adults. Circulation.

[18] Wahl PM, Rodgers K, Schneeweiss S, Gage BF, Butler J, Wilmer C, Nash M, Esper G, Gitlin N, Osborn N, Short LJ, Bohn RL (2010). Validation of claims-based diagnostic and procedure codes for cardiovascular and gastrointestinal serious adverse events in a commercially-insured population. Pharmacoepidemiol Drug Saf.

[19] Watson DJ, Harper SE, Zhao PL, Quan H, Bolognese JA, Simon TJ (2000). Gastrointestinal tolerability of the selective cyclooxygenase-2 (COX-2) inhibitor rofecoxib compared with nonselective COX-1 and COX-2 inhibitors in osteoarthritis. Arch Intern Med.

[20] Moore RA, Derry S, Makinson GT, Mcquay HJ (2005). Tolerability and adverse events in clinical trials of celecoxib in osteoarthritis and rheumatoid arthritis: systematic review and meta-analysis of information from company clinical trial reports. Arthritis Res Ther.

[21] Silverstein FE, Faich G, Goldstein JL, Simon LS, Pincus T, Whelton A, Stenson WF, Burr AM, Zhao WW, Kent JD, Lefkowith JB, Geis GS. Gastrointestinal toxicity with celecoxib versus nonsteroidal anti-inflammatory drugs for osteoarthritis and rheumatoid arthritis. J Am Med Assoc. 2000;284:1247–55. 10.1001/jama.284.10.1247 10979111

[22] Bombardier C, Laine L, Reicin A, Shapiro D, Burgos-Vargas R, Davis B, Day R, Ferraz MB, Hawkey CJ, Hochberg M, Kvein TK, Schnitzer T, Hospital MS, De Medicina EP, Sciences S, Hospital D (2000). Comparison of upper gastrointestinal toxicity of rofecoxib and naproxen in patients with rheumatoid arthritis. N Engl J Med.

[23] Pisters R, Lane DA, Nieuwlaat R, de Vos CB, Crijns HJGM, Lip GYH (2010). A novel user-friendly score (HAS-BLED) to assess 1-year risk of major bleeding in patients with atrial fibrillation: the Euro Heart Survey. Chest.

[24] Gagne JJ, Glynn RJ, Avorn J, Levin R, Schneeweiss S (2011). A combined comorbidity score predicted mortality in elderly patients better than existing scores. J Clin Epidemiol.

[25] Friedman J, Hastie T, Tibshirani R (2010). Regularization paths for generalized linear models via coordinate descent. J Stat Softw.

[26] Arbogast PG, Ray WA (2011). Performance of disease risk scores, propensity scores, and traditional multivariable outcome regression in the presence of multiple confounders. Am J Epidemiol.

[27] Arbogast PG, Ray WA (2009). Use of disease risk scores in pharmacoepidemiologic studies. Stat Methods Med Res.

[28] Hansen BB. Bias reduction in observational studies via prognosis scores. In: Tech Rep #441 Stat Dept Univ Michigan. 2006. p. 1–29.

[29] Francis Cook E, Goldman L (1989). Performance of tests of significance based on stratification by a multivariate confounder score or by a propensity score. J Clin Epidemiol.

[30] Stürmer T, Schneeweiss S, Brookhart MA, Rothman KJ, Avorn J, Glynn RJ (2005). Analytic strategies to adjust confounding using exposure propensity scores and disease risk scores: nonsteroidal antiinflammatory drugs and short-term mortality in the elderly. Am J Epidemiol.

[31] Leacy FP, Stuart EA (2013). On the joint use of propensity and prognostic scores in estimation of the average treatment effect on the treated: a simulation study. Stat Med.

