# Supplementary material for: Dimension reduction and shrinkage methods for high dimensional disease risk scores in historical data
Source: Emerg Themes Epidemiol. 2016 Apr 5;13:5. doi: 10.1186/s12982-016-0047-x (PMC4822311; doi:10.1186/s12982-016-0047-x)
Supplement: Supplementary file 1 — 10.1186/s12982-016-0047-x Tables for codes used and predefined predictive factors for the two studies. [file 12982_2016_47_MOESM1_ESM.pdf]

Appendix Table I. Claims Codes Used to Identify Patients With Valvulopathy

Diseases of mitral valve	ICD9 diagnosis 394.x
Diseases of aortic valve	ICD9 diagnosis 395.x
Diseases of mitral and aortic valve	ICD9 diagnosis 396.x
Diseases of endocardial structures	ICD9 diagnosis 397.x
Other and unspecified rheumatic heart diseases	ICD9 diagnosis 398.9
Heart valve replaced by transplant	ICD9 diagnosis V42.2
Heart valve replaced by a mechanical device / prosthesis	ICD9 diagnosis V43.3
Open heart valvuloplasty without replacement	ICD9 procedure 35.1x
Replacement of heart valve	ICD9 procedure 35.2x
Atrioventricular valve repair	CPT 33660-33665
Aortic valve valvuloplasty	CPT 33400-33403
Mitral valve repair / valvuloplasty / replacement	CPT 33420-33430
Valvectomy, tricuspid valve, with cardiopulmonary bypass	CPT 33460
Tricuspic valve repair / valvuloplasty / replacement	CPT 33463-33468
Pulmonary valve replacement	CPT 33475
Prosthetic valve dysfunction repair	CPT 33496
Implantation of catheter-delivered prosthetic aortic heart valve	CPT 0257T
Transthoracic cardiac exposure for catheter-delivered aortic valve;	CPT 0258T, 0259T
Implantation of catheter-delivered prosthetic pulmonary valve	CPT 0262T

Appendix Table II.

Hospital Discharge Codes For Identification of Major Hemorrhages for Dabigatran Study

	Diagnoses	Code
Major Intracranial Bleed	Intracranial hemorrhage	430.x
	Intracerebral hemorrhage	431.x
	Other and unspecified intracranial hemorrhage	432.x
Major Gastro-intestinal Bleed	Gastric ulcer with hemorrhage	531.0x, 2x, 4x, 6x
	Duodenal ulcer with hemorrhage	532.0x, 2x, 4x, 6x
	Peptic ulcer, site unspecified, with hemorrhage	533.0x, 2x, 4x, 6x
	Gastrojejunal ulcer with hemorrhage	534.0x, 2x, 4x, 6x
	Diverticula of small intestine with hemorrhage	562.02, 562.03
	Diverticula of colon with hemorrhage	562.12, 13
	Hemorrhage of rectum and anus	569.3x
	Gastrointestinal hemorrhage	578.x
	Procedures codes for endoscopic control of gastric or duodenal bleeding, or upper gastrointestinal endoscopy with control of bleeding	ICD-9 44.43, CPT code 43225
Major urogenital bleed	Hematuria	599.7
	Excessive or frequent menstruation (with anemia)	626.2 with 280.0, 285.1, 285.9
Other major bleeds	Hemarthrosis	719.1x
	Hemopericardium	423.0
	Hemoptysis, Epistaxis	786.3x, 784.7x
	Acute posthemorrhagic anemia	285.1x
	Hemorrhage, unspecified	459.0x

Appendix Table III.

A) Predefined Predictive Factors of Gastrointestinal Bleeding For the Coxib Study

Comorbidities	Chronic heart failure
	Hypertension
	Recent myocardial infarction
	Old myocardial infarction
	Peripheral vascular disease
	Prior stroke
	Prior transient ischemic attack
	Chronic kidney disease
	Osteoarthritis
	Rheumatoid arthritis
	Prior gastrointestinal disease
Concurrent medication	Warfarin
	Gastroprotective agents
	Clopidogrel
	Corticocolticoid
Health services use	Hospital admission
	Nursing home stays
	Number of MD visits
	Number of Medications used

B) Predefined Predictive Factors of Major Hemorrhagic Events for the Dabigatran Study

Comorbidities	Chronic kidney disease
	Hypertension
	Gastrointestinal bleeding (hospitalization)
	Gastrointestinal bleeding (outpatient diagnosis)
	Peripheral artery disease
	Anemia
	Chronic liver disease
	Alcohol abuse
	Drug abuse
Concurrent medication	nsNSAIDs / coxibs
	Antiplatelets
