# Supplementary material for: Dimension reduction and shrinkage methods for high dimensional disease risk scores in historical data
Source: Emerg Themes Epidemiol. 2016 Apr 5;13:5. doi: 10.1186/s12982-016-0047-x (PMC4822311; doi:10.1186/s12982-016-0047-x)
Supplement: Supplementary file 2 — 10.1186/s12982-016-0047-x Calibration Plots for Selected Models from dabigatran study. [file 12982_2016_47_MOESM2_ESM.pdf]

Appendix eFigure.

Calibration Plots for Selected Models from dabigatran study

Demographics + HASBLED score [model 2]

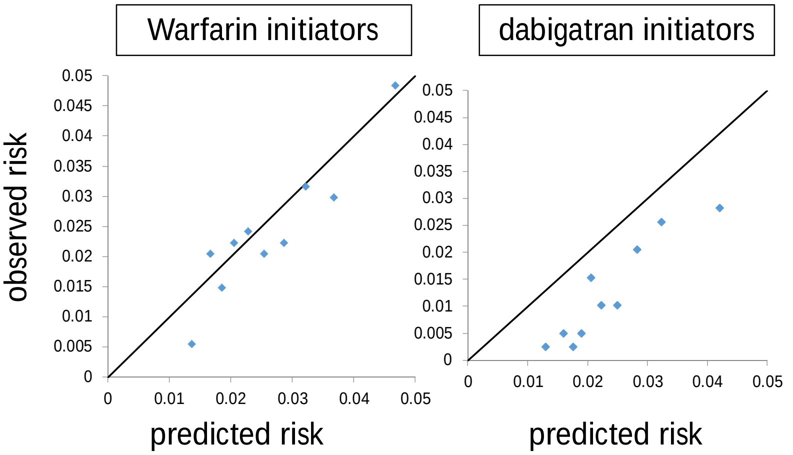

C-stats: 0.62
HL: 5.6 (p-value=0.69)

C-stats: 0.70
HL: 24.6 (p-value<.01)

Demographics +500 empirical covariates [model 4]

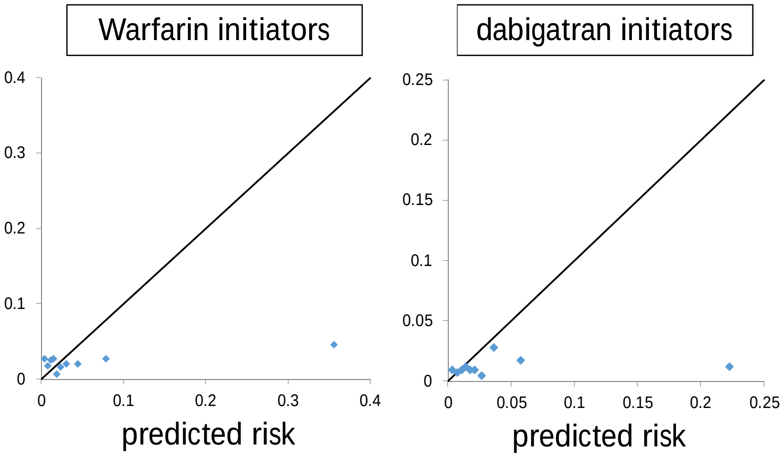

C-stats: 0.54
HL: 384 (p-value<.01)

C-stats: 0.56
HL: 126 (p-value<.01)

Demographics + PCA(10) [model 5]

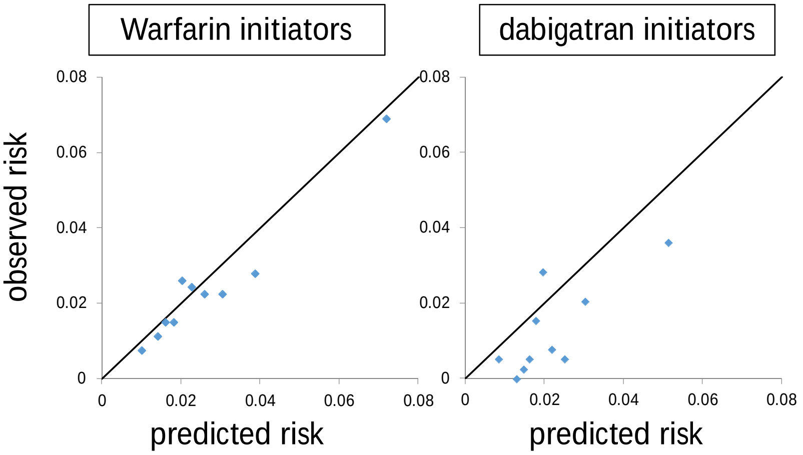

C-stats: 0.66
HL: 5.2 (p-value=0.73)

C-stats: 0.68
HL: 27.0 (p-value<.01)

lasso(demographics + score + predefined variables +PCA(30)) [model 14]

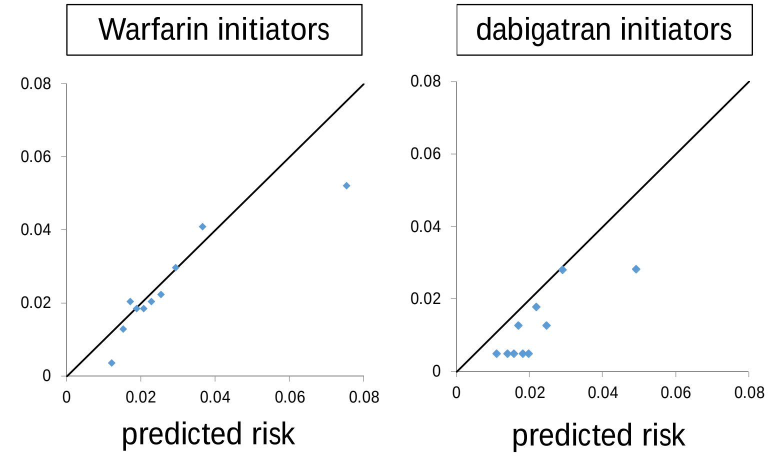

C-stats: 0.65
HL: 10.7 (p-value=0.22)

C-stats: 0.67
HL: 19.5 (p-value=.01)
